# Cholera diagnosis in human stool and detection in water: protocol for a systematic review of available technologies

**DOI:** 10.1186/s13643-018-0679-8

**Published:** 2018-02-20

**Authors:** Karin Diaconu, Jennifer Falconer, Fiona O’May, Miguel Jimenez, Joe Matragrano, Betty Njanpop-Lafourcade, Alastair Ager

**Affiliations:** 1 Institute for Global Health and Development, Queen Margaret University - Edinburgh, Musselburgh, EH21 6UU Scotland; 2 Koch Institute for Integrative Cancer Research, Massachusetts Institute of Technology, Cambridge, MA 02139 USA; 3 Columbia University, 116th St & Broadway, New York, NY 10027 USA; 4 Agence de Médecine Préventive, 21 boulevard Pasteur, 75015 Paris, France; 5 Mailman School of Public Health, 116th St & Broadway, New York, NY 10027 USA

**Keywords:** Cholera, Diagnosis, Diagnostic product, Technology, Medical device, Cholera surveillance, Cholera infection prevention and control

## Abstract

**Background:**

Cholera is a highly infectious diarrheal disease spread via fecal contamination of water and food sources; it is endemic in parts of Africa and Asia and recent outbreaks have been reported in Haiti, the Zambia and Democratic Republic of the Congo. If left untreated, the disease can be fatal in less than 24 h and result in case fatality ratios of 30–50%.

Cholera disproportionately affects those living in areas with poor access to water and sanitation: the long-term public health response is focused on improving water and hygiene facilities and access. Short-term measures for infection prevention and control, and disease characterization and surveillance, are impaired by diagnostic delays: culture methods are slow and rely on the availability of infrastructure and specialist equipment. Rapid diagnostic tests have shown promise under field conditions and further innovations in this area have been proposed.

**Methods:**

This paper is the protocol for a systematic review focused on identifying current technologies and methods used for cholera diagnosis in stool, and detection in water. We will synthesize and appraise information on product technical specifications, accuracy and design features in order to inform infection prevention and control and innovation development.

Embase, MEDLINE, CINAHL, Proquest, IndMed and the WHO and Campbell libraries will be searched. We will include studies reporting on field evaluations, including within-study comparisons against a reference standard, and laboratory evaluations reporting on product validation against field stool or water samples. We will extract data according to protocol and attempt meta-analyses if appropriate given data availability and quality.

**Discussion:**

The systematic review builds on a previous scoping review in this field and expands upon this by synthesising data on both product technical characteristics and design features. The review will be of particular value to stakeholders engaged in diagnostic procurement and manufacturers interested in developing cholera or diarrheal disease diagnostics.

**Systematic review registration:**

PROSPERO CRD42016048428.

**Electronic supplementary material:**

The online version of this article (10.1186/s13643-018-0679-8) contains supplementary material, which is available to authorized users.

## Background

One hundred sixty years have passed since John Snow identified the Broad Street water pump as the source of the 1864 London cholera outbreak [1]. Today, cholera is largely absent across high-income countries. However, the disease persists in low-resource and humanitarian settings where it disproportionately affects those living in overcrowded areas with poor or no access to clean water and sanitation [2–4]. In 2015 alone, the World Health Organization (WHO) recorded 172,454 cholera cases and 1304 deaths; 41% of these were reported in Africa, with a further 37 and 21% respectively originating in Asia and the Americas [3]. The aforementioned estimates are a likely underestimation of the true toll of the disease: modelling exercises predict 1.4 to 4.0 million cholera cases occur worldwide annually, resulting in approximately 21–143,000 deaths [5]. Cholera is endemic in many parts of Africa [6], and recent outbreaks have been reported in the Democratic Republic of the Congo, Zambia, the Dominican Republic and Haiti as well as Central and West Africa [2, 3, 7].

Cholera is a highly infectious diarrheal disease caused by the *Vibrio cholerae* O1 and 139 bacteria [8]. Adults and children are equally susceptible and become ill upon ingesting contaminated foods or water [6, 9]. Symptoms of affected patients include severe dehydration, vomiting and characteristic “rice water stool”; if left untreated, the disease can be fatal in less than 24 h leading to high population level case fatality rates (30–50%) [9]. While treatment is inexpensive and easy to administer—it consists of the administration of oral rehydration solution and rest, cholera infection prevention and control (IPC) and surveillance are not [6, 8–11]. Two issues are of particular relevance in this regard.

First, 80% of cholera-affected patients do not exhibit typical symptoms; infected persons may therefore unknowingly propagate the spread of the disease [3, 9]. Cholera bacteria can live up to 4 weeks in an infected persons’ stool, spreading to food sources or contaminating the water supply of communities [9].

Second, diagnosis is costly and disease and outbreak confirmation rely on the presence of laboratory infrastructure [8, 9, 12]. Culture methods are the current diagnostic gold standard [13], but prove to be time-consuming (more than 24 h) and assume the presence of suitable laboratory equipment, reagents and human resources skilled in performing appropriate tests [8]. As laboratories and skilled technicians are in short supply in humanitarian and low-resource settings, rapid diagnostic tests (RDTs) ready for immediate field deployment have been developed [10, 12]. RDTs appear increasingly fast and easy to use, but are prohibitively expensive and insufficiently sensitive and specific to aid in cholera detection (versus diarrhoeal diseases) [8, 13].

Timely cholera IPC and improved surveillance are predicated on the availability, and procurement, of inexpensive and accurate diagnostic products [6, 10, 11, 14]. To inform product development efforts, as well as offer evidence to stakeholders engaged in cholera diagnostic product procurement, up-to-date information on the comparative accuracy, technical specification and design of currently available technologies is needed.

## Methods and systematic review design

### Study design and scope

This paper serves as a protocol for a systematic review that aims to identify any relevant cholera diagnosis and detection products for use in either stool or water globally and appraise the ancillary evidence base.

### Review objectives

In line with study questions (Table 1), the review seeks to:

1. Document technical specifications of cholera diagnosis/detection products and appraise the ancillary evidence base, by:
  - Identifying products currently used (or about to be commercially available) for cholera diagnosis in stool and detection in water;
  - Synthesising information on product technical characteristics (e.g. detection target and limit, accuracy, reliability);
  - Reviewing the sensitivity and specificity of available testing products, if appropriate given data quality, synthesising quantitative information on test performance in a meta-analysis;
  - Critically appraising the quality of studies forming the evidence base for each diagnostic product.
2. To provide a structured narrative account of issues arising in product deployment and use, by:
  - Extracting quotes from included studies on product pricing, availability, design features (e.g. ease of use) and accounts of product use;
  - Coding, thematically grouping and iteratively interpreting the data to identify how/why certain products are comparatively more sustainable or successfully deployed for cholera IPC or surveillance.

**Table 1:** Review questions

The systematic review we propose addresses the following research questions:
1. What cholera diagnosis and detection products are currently available?
2. How do the above-identified products perform with respect to their specificity, sensitivity and accuracy?
3. What challenges arise in the deployment and use of current diagnostic products and detection methods?
4. What design characteristics should products have to enable improved cholera IPC and surveillance?

### Scoping searches

Scoping searches on the review topic were conducted in August 2016 and indicate a dearth of review materials available relating to cholera diagnosis. Searches included PROSPERO, the Cochrane Library and MEDLINE; the former databases were searched using the term “cholera”; in MEDLINE, the terms “diagnosis” or “detection” were added.

PROSPERO indicated five systematic reviews on cholera are on-going [15–19]; topics ranged from the safety and effectiveness of antibacterial treatment [15] to the effectiveness of oral cholera vaccine when used reactively [18]. The Cochrane Library held records of eight Cochrane [20–27] and four Centre for Reviews and Dissemination (CRD) reviews of similar focus to PROSPERO protocols [28–31]; documents explored the effects and impact of vaccines and chemoprophylaxis [26, 27, 29, 31], antimicrobial and antibiotic treatment [25, 31] and oral rehydration salts [23, 28].

A review of titles in MEDLINE identified a journal article of particular relevance by Dick et al. [12]. In this review, the authors search the English language bibliographic and grey literature since 1990 and identify and describe the technical specifications of 24 commercially available diagnostic products available for use in low-resource and humanitarian settings [12]. The authors rank diagnostic products that have been evaluated in field accuracy studies according to positive and negative predicted values; five rapid diagnostics are identified as showing particular promise for further use in IPC/surveillance (COAT, Institute Pasteur Cholera Dipstick, SMART, Institute Pasteur Dipstick and Medicos). Dick et al. recommend the latter products for further evaluation via independent field studies [12].

While the review by Dick et al. offers a comprehensive starting point for research on cholera diagnostics, the review was not systematic and is likely to have overlooked studies of relevance reported outwith English. Additionally, minimal details on the processes of review management, study selection and data extraction are noted: it is unclear who conducted searches, selected studies and how data were extracted, aggregated and primary studies appraised. Notably, the scoping review synthesises data on product technical specifications (i.e. sensitivity/specificity, turn-around time), but neglects to present information on product design (e.g. ease of use, training requirements) or pricing (e.g. price per test and cost per test-use). Information on the latter issues is highly pertinent given the variability in available human resources to carry out cholera IPC/surveillance, high unit price of rapid diagnostic tests and limited infrastructure to support laboratory-based diagnosis confirmation [8, 9, 12].

### Search strategy and information sources

**Fig. 1:**
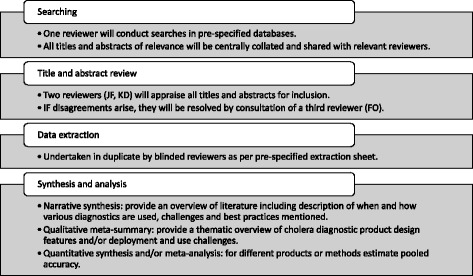
Systematic review process. This figure provides details on the search, abstract review, data extraction and synthesis and analysis processes of the review

We will search MEDLINE, CINAHL, Embase, IndMed, Scopus, Proquest, the Campbell collaboration and the WHO libraries (WHOLIS) (see Fig. 1). Tables 2 and 3 illustrate search strings to be used for MEDLINE and CINAHL; similar searches will be conducted in the remaining databases listed and studies selected as per criteria outlined below. The Cochrane Library has been excluded from searches as no trials or articles of relevance on cholera diagnostic products or methods were identified during scoping searches. In addition to the above sources, we will search reference lists from relevant articles and include the studies identified by Dick et al. [12] We will also search grey literature (e.g. OpenGrey).

**Table 2:** Search terms used to search MEDLINE

S1 (MH “Cholera”) OR “cholera” OR (MH “Cholera Toxin”)	
S2 (MH “Diagnosis”) OR “diagnosis” OR (MH “Early Diagnosis”) OR (MH “Diagnosis, Differential”)	
S3 (MH “Limit of Detection”) OR “detection”	
S4 (MH “Diarrhea+”) OR “diarrhoea”	
S5 (MH “Water”) OR “water” OR (MH “Drinking Water”) OR (MH “Water Pollution”) OR (MH “Water Wells”) OR (MH “Water Supply”) OR (MH “Water Quality”) OR (MH “Waste Water”)	
S6 AB choler*	
S7 AB diagnos* OR AB detect*	
S8 AB stool OR AB diarrh* OR AB water	
S9 S2 OR S3	
S10 S4 OR S5	
S11 S1 AND S9 AND S10	
S12 S6 AND S7 AND S8	
S13 S11 OR S12	
S14 Limit S13 to (yr = “1990-current”)

**Table 3:** Search string used to search CINAHL

S1 AB choler*
S2 AB diagnos* OR AB detect*
S3 AB stool OR AB fec* OR AB faec* OR AB diarrh* OR AB water
S4 S1 AND S2 AND S3
S5 (MH “Cholera”) OR “cholera”
S6 (MH “Diagnosis”) OR “diagnosis” OR (MH “Diagnosis, Laboratory”) OR (MH “Early Diagnosis”)
S7 (MH “Diarrhea”) OR “diarrhoea”
S8 (MH “Feces”) OR “feces”
S9 stool
S10 (MH “Water”) OR (MH “Water Pollution”) OR (MH “Water Supply”) OR (MH “Water Microbiology”)
S11 S2 OR S6
S12 S7 OR S8 OR S9 OR S10
S13 S5 AND S11 AND S12
S14 S4 OR S13
S14 Limit S13 to Jan 1990-Sept 2017

### Study and data management

KD will act as main reviewer and data custodian. The study will be conducted in line with the CRD’s guidance on diagnostic accuracy systematic reviews [32].

Two reviewers (JF, KD) will screen all titles and abstracts for inclusion. Articles identified will be downloaded directly into EndNote/Zotero for screening. Data extraction will be conducted by two reviewers, in duplicate and blinded as per standard systematic review methodology. Information on pre-specified items will be extracted into a Microsoft Excel document (see the “Data extraction” section below and Additional file 1). Following resolution of disagreements, a collated version of extracted data will be made available to the review team for analysis.

Disagreements between reviewers will be resolved by consultation of a third reviewer (FO).

### Study selection

**Fig. 2:**
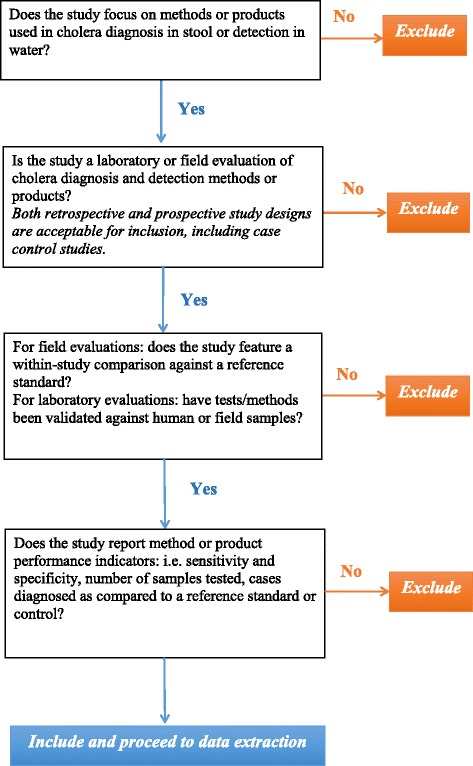
Selection criteria applied to abstracts retrieved via literature searches. This figure is a decision tree outlining how study selection criteria will be applied during abstract screening

Figure 2 outlines the study selection and data extraction procedures employed. We will include studies from any global settings reporting on any diagnostic technologies targeting cholera O1 or O139, of both classical and El Tor biotypes and Ogawa and Inaba serotypes [33].

We will include primary diagnostic accuracy studies (laboratory or field based) that focus on the evaluation of methods or products used in cholera diagnosis in human stool or detection in water. Prospective studies and studies retrospectively evaluating diagnostic or detection products will be included. We acknowledge that culture methods are considered the gold standard for cholera diagnosis and detection; however, we will include any studies reporting on the performance of index tests against a specified comparator test. To be included, studies must report the sensitivity and specificity of evaluated diagnostic products/methods.

No language restrictions will be applied; should studies outwith English be retrieved, the author group will commit to consulting translators to extract relevant data. Only articles beyond 1990 will be reviewed; this reflects the time of significant medical device and product development advances in the field of cholera diagnosis as reported by Dick et al. [12].

### Data extraction

Full texts of included abstracts will be retrieved and data extracted as per the pre-specified template (Additional file 1). The template has been tailored to the current study and closely mirrors the Cochrane group’s data extraction template for systematic reviews [34].

We will extract data on the following domains and items among others:

- Study characteristics: study design, duration and aims, populations included and sample characteristics, products evaluated, outcomes noted and statistical methods used;
- Diagnostic product or method characteristics: index and/or reference product name, technical specifications (e.g. test format, turn-around time, disease target and stability of test under transport and storage conditions), cost (e.g. price per test and cost per use of test), product design and use (e.g. handling, user training, availability in deployment settings).
- Data specific to reference and index tests mentioned: the data extraction sheet has been tailored to the specific type of accuracy study conducted, either laboratory or field based. For both reference and index tests, data will be extracted on diagnostic method description, test content and ancillary diagnostic procedures undertaken, economic or resource requirements of testing, test accuracy metrics (e.g. sensitivity, specificity, negative and positive predictive values).

### Data synthesis

We will use three methods for synthesis of extracted data (see Fig. 1):

- Narrative and quantitative synthesis

All studies, irrespective of quality, will be included in a narrative synthesis. As per Popay et al. [32, 35], we will synthesise insights from the reviewed literature in order to provide a theory of how cholera diagnostic products are used, including an account of deployment challenges and best practices. We will group, describe and critically appraise the studies and products noted therein according to study type, setting and population characteristics, and type of diagnostic/detection product or method. Should meta-analyses not be appropriate (see the below section), we will synthesise quantitative data and provide a descriptive account of diagnostic method and accuracy metrics.

- Meta-analyses

All texts will undergo screening for inclusion in meta-analysis. We anticipate conducting meta-analyses only for field evaluation studies that use culture methods as the reference standard for cholera detection (see Additional file 2). We will use sensitivity analyses to explore the effect of study quality on meta-estimates. Meta-analyses will be organized in sub-groups depending on the diagnostic product or method type evaluated, e.g. dipstick tests targeting lipopolysaccharides vs. culture methods. Meta-estimates of test sensitivity and specificity will be calculated; we will additionally calculate *I*^2^ statistics. Should a substantial number of studies report diagnostic accuracy in comparison to reference standards other than culture, a further meta-analysis may be conducted.

We will follow the guidance of the Cochrane group when conducting meta-analyses [36].

- Qualitative meta-summary

For extraction items focused on capturing product design characteristics, reviewers will extract quotes from appraised documents. The specific data items will serve as the basis of a deductive coding framework. Reviewers will extract any additional quotes of relevance in a further “other” named extraction column; the latter will then undergo inductive coding. All quotes will form part of a thematic analysis and qualitative meta-summary as per Sandelowski et al. [37].

### Assessment of risk of bias and quality of evidence

We will assess the risk of bias in individual studies via use of the QUADAS2 tool [38]—tailored specifically to study purposes and included as a separate worksheet in the data extraction template (Additional file 1). In addition to this, we will assess confidence in cumulative evidence by following a GRADE approach [39].

### Reporting

We will follow PRISMA reporting guidelines [40] and enclose a PRISMA-P checklist as an Additional file 2.

## Discussion

We propose to undertake a systematic review of studies evaluating the accuracy, design and availability/pricing of products for cholera diagnosis in stool or detection in water. The review is particularly timely given recent international efforts in cholera surveillance and the specified need for improved diagnostic capacity [41]. The systematic review will assist both health care professionals engaged in selecting diagnostic devices for procurement and manufacturers and researchers engaged in cholera-specific diagnostic product development.

We acknowledge several limitations. First, search terms are broad and may retrieve a particularly large number of studies; materials retrieved are likely to be highly heterogeneous, spanning both laboratory and field evaluations of cholera diagnostic and detection products. In line with Dick et al. [12], we note that this is necessary in order for the review to capture up-to-date information on all diagnostic products available for supporting cholera IPC/surveillance. To standardize data extraction and ensure we systematically appraise evidence, we have tailored data extraction to study type (either field or laboratory) and will report on all extracted data in detail in line with the GRADE approach [39].

Second, we are restricting inclusion to laboratory studies validating index tests against field samples and to field evaluations reporting within-study comparisons against a reference test. This exclusion policy is designed to capture information only on products most likely to proceed to use in the field in the near future. This implies that proof-of-concept or incipient evaluation studies reporting on innovative product development will be excluded. To still capture information on products in early stages of development, we will produce detailed tables noting studies excluded from our analysis.

Third, we acknowledge that meta-analyses may not be possible due to a restricted and low-quality evidence base in this area. In particular, scoping searches suggest studies may omit reporting population characteristics, confounders and diagnostic thresholds. Dick et al. additionally note that diagnostic evaluations are frequently conducted by product developers themselves, potentially biasing reporting and study design [12]. We will rigorously assess the risk of bias within studies and across the body of evidence in order to assess the appropriateness of conducting a meta-analysis.

Despite the above limitations, we emphasize the systematic review proposed here is of particular research and practical value. The study aims to offer a comprehensive, up-to-date account of products currently (or soon to be) available for cholera diagnosis in low-resource settings. In contrast to previous work, we seek to describe and synthesise information not only on product technical characteristics but also design features and notes on product availability/pricing. This will be of particular use to stakeholders directly engaged in cholera IPC and surveillance when selecting diagnostic products for investment and use in cholera outbreaks

## Additional files

Additional file 1: Template for data extraction. (XLSX 22KB)

Additional file 2: PRISMA-P (Preferred Reporting Items for Systematic review and Meta-Analysis Protocols) 2015 checklist: recommended items to address in a systematic review protocol. (DOCX 17 kb)

## Abbreviations

CINAHL: Cumulative Index to Nursing and Allied Health Literature
COAT: Coagglutination Test
CRD: Centre for Reviews and Dissemination
GRADE: Grading of Recommendations Assessment, Development and Evaluation
IPC: Infection prevention and control
PRISMA: Preferred Reporting Items for Systematic Reviews and Meta-Analyses
PROSPERO: International prospective register of systematic reviews
QUADAS: Quality Assessment of Diagnostic Accuracy Studies
RDT: Rapid Diagnostic Test
SMART: Sensitive Membrane Antigen Rapid Test
WHO: World Health Organization
